# Factor structure of the Utrecht Work Engagement Scale in a sample of Danish and Swedish haemodialysis nurses

**DOI:** 10.1186/s12912-025-03545-4

**Published:** 2025-07-10

**Authors:** Maria Lindberg, Kati Knudsen, Magnus Lindberg

**Affiliations:** 1 Faculty of Health and Occupational Studies, Department of Health and Caring Sciences, University of Gävle, Gävle, SE 801 76 Sweden; 2 Centre for Research and Development, Region Gävleborg/Uppsala University, Gävle, SE 80188 Sweden

**Keywords:** Psychometric evaluation, UWES, Work engagement

## Abstract

**Background:**

The Utrecht Work Engagement Scale is a frequently used instrument for measuring work engagement. However, the instrument’s conceptual underpinnings, theoretical clarity and dimensional structure has been criticized, and its construct validity has not been evaluated appropriately in samples of nurses. The study purpose was to assess the psychometric properties of the Utrecht Work Engagement 9-item Scale (UWES-9) in a sample from the haemodialysis nurse population using approaches that take the ordinal nature of the data into account.

**Methods:**

The psychometric properties were evaluated in accordance with classical test theory focusing on data completeness, unidimensionality, scaling assumptions, targeting and reliability. The analyses were conducted using data derived from a cross sectional study.

**Results:**

Missing item responses were < 2%. Exploratory factor analysis based on polychoric correlations and parallel analysis identified one factor. Scaling assumptions were supported based on desirable levels of corrected item-total correlations. The targeting analyses displayed expected score distribution, no floor effect and acceptable ceiling effect. However, the mean total score was above the scale midpoint. The reliability, estimated using Ordinal alpha, was 0.93.

**Conclusions:**

The study findings, evaluated using methods that consider the ordinal nature of the scale ratings, indicate that the UWES-9 is a reliable and valid unidimensional measure with high data completeness, strong internal consistency, and a supported factor structure. These results support the use of the UWES-9 in research and practice for assessing work engagement. However, further studies are recommended to examine its applicability across diverse populations and settings.

**Trial registration:**

Not applicable.

## Introduction

The concept of work engagement has been developed to emphasize the positive rather than the negative side of workers’ well-being [1]. The principal definition of work engagement is “*a positive*,* fulfilling*,* work-related state of mind that is characterised by vigour*,* dedication*,* and absorption*” [2, page 74]. Vigour deals with having the energy and will to put effort into one´s work and persist when dealing with challenges. Dedication concerns having significant involvement and identification with the job and includes being inspired and proud of one’s own work. Absorption refers to the ability to be fully engrossed in one’s work, so that time passes quickly, and one has difficulties detaching oneself from work [1]. In their literature review, Garcia-Sierra and colleagues [3] concluded that nurses’ work engagement has a direct impact on healthcare outcomes. The level of work engagement among nurses is modifiable [4, 5], and engaging leadership is a prominent factor [6] in making nurse managers key players in the promotion of work engagement. The work climate and support from the work organisation are other influencing factors in relation to the promotion of nurses’ work engagement [3].

The single most popular and widely used instrument for measuring work engagement in research is the Utrecht Work Engagement Scale (UWES). The UWES was originally developed in 2002 as a 17-item, self-administered questionnaire comprising three subscales designed to measure Vigour, Dedication and Absorption [2]. The original 17-item version has been shortened over time in several stages, and there are now officially 15-item, 9-item [7] and 3-item version [8] of the UWES available. Notably, in nursing research, versions of the UWES other than the official have also been used, including 16-item, 14-item and 11-item adaptations [3], which may further complicate assessments of its factorial validity. While the UWES has evolved through multiple iterations, questions regarding its psychometric robustness have persisted.

Despite its wide-spread use, the UWES has attracted some criticism. Mills et al. [9] argued that the methodology employed in the development of the original scale was flawed in respect to the establishment of its factorial validity, referring to the extent to which the scale’s factor structure aligns with theoretical expectations. Further criticism has been directed at the factor structure of the instrument, particularly the close correlations observed among the three subscales – Vigor, Dedication and Absorption – raising questions regarding the superiority of the 3-factor structure over a 1-factor structure based solely on the total scale score. Supporting this concern, several studies have failed to confirm the 3-factor structure in their samples (for example., 10,11). Kulikowski [12] investigated the factorial validity of the UWES in a literature review and found that, of the 11 studies investigating the UWES-9 alone, three confirmed the 1-factor structure, three the 3-factor structure, four studies found the two structures to be equivalent, and one study failed to support either alternative. Accordingly, Kulikowski concluded that no definitive recommendations could be made based on the review.

As most classical-test-theory-based psychometric studies on UWES are performed using parametric analyses [3, 10–12], they do not account for the fact that item-level data are ordinal. Thus, it can be stated that the optimal factorial structure of the UWES remains unknown, despite the considerable number of studies. Moreover, studies conducted among nurses (for example., 13–15) have mostly relied on the original validation work, and such reliance poses threats to the statistical conclusion validity and construct validity of these studies. It is important that the UWES be evaluated on the structure that best suits the respective populations using polychoric-based correlation and reliability estimates, as these diminish the bias associated with parametric procedures applied to ordinal data [16]. Because nurses’ working conditions differ across specialties, it is important to also take this phenomenon into account when evaluating the reliability of scales. Nurses who care for people with kidney failure need to have specific competencies [17], and the work environment at haemodialysis centres is considered demanding, with both high mental [18, 19] and high physical workload that negatively impact on their health [20, 21]. Using a shorter version of an established instrument, such as UWES-9 instead of UWES-17, can be justified on practical and methodological grounds. A shorter scale reduces response burden, increases completion rates, and facilitates repeated measurements in longitudinal studies. It minimizes redundancy, enhances data quality by reducing survey fatigue and response bias. If empirical evidence supports it, a shorter version is a more efficient alternative without compromising measurement precision. Considering this, the aim of the present study was to assess the psychometric properties (structural validity and internal consistency) of the Utrecht Work Engagement 9-item Scale (UWES-9), in a sample from the haemodialysis nurse population, using approaches that take the ordinal nature of the data into account.

## Methods

### Design

This psychometric evaluation was embedded in a cross-sectional study [21].

### Sample

Self-rated estimates of work engagement were taken from a web-based survey. An invitation to participate in the survey, including a link, was sent via e-mail to the entire population of 1023 nurses on duty at the 47 haemodialysis centres included in Denmark and Sweden [21]. Around half (53.2%) of the considered nurses responded to the UWES-9 and were thus included in this study. Approximately one-third of the participants worked at a dialysis centre in Denmark, while two-thirds were drawn from Swedish dialysis centres. Among the participants, 94.1% were women, and on average, they had 11.5 (SD 9.2, range 0–42) years of experience in dialysis care.

### Measure

The UWES-9 is for non-commercial research freely available on Schaufeli’s website (https://www.wilmarschaufeli.nl/downloads/); we used the existing translations into Danish and Swedish, respectively. The UWES-9 is a self-administered scale composed of nine items answered on a 7-point frequency scale ranging from 0 to 6 [7]. The response scale is ordinal [22], as the wording of the response alternatives (“0 Never / 1 Almost Never = A few times a year or less / 2 Rarely = Once a month or less / 3 Sometimes = A few times a month / 4 Often = Once a week / 5 Very often = A few times a week / 6 Always = Every day) cannot be assumed to have equivalent intervals between them. All 9-item scores are added and then averaged into a total score ranging from 0–6. According to the manual, three partial scores are also added, and then the sum is divided by the number of items composing each subscale (Vigour, Item 1, 2, 5; Dedication, Item 3, 4, 7; and Absorption, Item 6, 8, 9). Higher scores indicate greater work engagement [7].

### Data analysis

The psychometric analyses of UWES-9 were conducted according to classical test theory [22] using IBM SPSS Statistics for Windows, Version 27.0. Armonk, NY: IBM Corp, FACTOR version 12.01.02, and R version 4.3.0 (psych package version 2.3.9) r-project.org. Data completeness was used as an indicator of scale acceptability among respondents and was assessed by calculating the percentage of missing responses to each item. Up to 5% missing item responses have been considered acceptable [22]. Scaling assumptions were tested to assess whether item scores can reasonably be averaged to a scale score by calculating item mean scores, standard deviations, and corrected item-total correlations. According to classical test theory principles, scaling assumptions are met if item means and standard deviations are roughly equal, and if corrected item-total correlations exceed 0.3 [23].

Given the lack of consensus in the literature as to the underlying factor structure of the UWES-9, exploratory factor analyses (EFA) were performed to examine the dimensionality, rather than a confirmatory approach. Moreover, to uncover latent constructs when dealing with ordinal data, we chose EFA based on a matrix of polychoric correlations because of its robustness against normality violations. The Kaiser-Meyer-Olkin (KMO) measure of sampling adequacy was used to ensure the conductibility of the EFA. A KMO value of 0.50 is generally regarded as the minimum threshold for proceeding with factor analysis; values below this indicate that the correlations among variables are insufficiently large to justify factor extraction [24]. Parallel analysis was used to determine the number of factors to retain. Based on the idea that real data with an underlying factor structure should produce factors with eigenvalues larger than those derived from random datasets with identical numbers of cases and variables, parallel analysis compares eigenvalues from real data with average eigenvalues from, in our case, 500 permutations. A factor is retained if its eigenvalue is larger than the parallel average eigenvalue. The three methods we utilized were: Optimal implementation of Parallel Analysis [25], Minimal Average Partial test (MAP) [26], and Hull method for selecting the number of common factors [27]. While all three methods aim to determine the optimal number of factors, they differ in their statistical approaches and the specific criteria they use for factor retention. Velicer’s MAP Test focuses on minimizing residuals, Hull’s Method compares eigenvalues with those derived from random data, and Timmerman and Lorenzo-Seva’s Parallel Analysis improves on traditional parallel analysis to increase precision. Despite these differences, each method contributes valuable tools to the toolkit of researchers seeking to perform factor analysis with a robust and statistically sound basis. For the analyses conducted in the FACTOR programme, missing values were identified using a numerical code and were automatically handled within the analysis algorithms. For the remaining descriptive analyses, the pairwise deletion approach was applied, allowing for the inclusion of all available data for each variable while excluding only the cases with missing values for specific variables. This method ensures maximal use of the dataset without compromising the integrity of the analysis.

Targeting was assessed using descriptive statistics to evaluate the distribution of UWES item ratings. This included examining the extent to which the ratings spanned the full range (0–6), had an average close to the scale midpoint and exhibited minimal skewness. Floor and ceiling effects were also analysed, with 15% considered acceptable [28]. Reliability was assessed using Ordinal alpha based on the polychoric correlation matrix, and therefore more accurately estimated internal consistency for measures of ordinal nature compared to the traditional Cronbach’s alpha [29].

## Results

Of the 544 nurses who answered the UWES-9, 520 completed all items. Thus, data completeness was considered good, with less than 2% missing item-level data. However, one item (Item 8 “I am immersed in my work”) excelled by having more missing values (*n* = 10) than the other items (range 2–5 missing). Correspondingly, the availability of computable total average scores was high. In the sample, item mean scores ranged from 3.97 to 5.01, and item SD ranged from 0.97 to 1.51 (Table 1). The assessed scaling assumptions were supported based on desirable levels of corrected item-total correlations (Tables 1 and 2).

The Kaiser-Meyer-Olkin measure of sample adequacy was good (KMO, 0.87), and the Bartlett’s test showed *p* < 0.001, which means that the conductibility of the EFA was supported. All subsequently conducted procedures for determining the number of factors and the EFA results provided support for the unidimensionality of the UWES-9. Specifically, all methods—Timmerman and Lorenzo-Seva’s Optimal Implementation of Parallel Analysis, Velicer’s MAP test, and the Hull method—consistently recommended retaining a single factor. In detail, Timmerman and Lorenzo-Seva’s Optimal Implementation of Parallel Analysis indicated that the second empirical factor in the EFA explained only 9.5% of the common variance compared to the 23.1% explained by the second factor derived from random data (Table 2). The Velicer’s MAP test showed an average partial of 0.0689 and this identified one dimension as the point at which the addition of further factors no longer leads to a significant reduction in the residual variance. Moreover, the Hull method determined one factor based on the comparison of observed eigenvalues with eigenvalues derived from random data and shoved a scree test value of 309.9. The unidimentional (1-factor) model explained 77.2% of the total variance, and the factor loadings ranged between 0.67 and 0.91. Targeting analyses (Table 2) indicated no floor effect, but there was a small (2.1%) yet acceptable ceiling effect observed in the total UWES-9 score. However, the mean total score for the UWES-9 was above the scale midpoint, and it exceeded 1 SD of the observed total scale mean score. Total UWES-9 score skewness was acceptable. The internal consistency estimated using Ordinal alpha was 0.93.

**Table 1:** Item score distribution, item endorsement frequency (*n* = 544), and factor loadings of the UWES-9 version (*n* = 520)

Item number	Median (q1-q3)	Mean (SD)	Item score distribution %							Missing data %	Factor loadings	Corrected polyserial item-total correlation
			0	1	2	3	4	5	6			
1	5 (4–5)	4.34 (1.2)	0.9	1.7	5.1	15.4	19.1	46.9	10.5	0.4	0.791	0.77
2	5 (4–5)	4.49 (1.0)	0.2	0.7	3.3	13.6	20.8	49.6	11.2	0.6	0.837	0.81
3	5 (4–5)	4.55 (1.1)	0.7	1.1	2.4	12.7	20.8	45.6	16.4	0.4	0.913	0.89
4	5 (4–5)	4.44 (1.2)	0.4	1.7	4.6	13.8	21.5	41.2	16.2	0.7	0.883	0.85
5	5 (4–5)	4.30 (1.3)	1.8	2.2	5.3	13.6	21.5	41.5	13.4	0.6	0.834	0.80
6	4 (3–5)	4.11 (1.3)	0.9	3.5	7.7	16.0	24.8	36.2	9.9	0.9	0.827	0.80
7	5 (5–6)	5.01 (1.0)	0	0.6	1.5	6.3	13.4	44.1	33.5	0.7	0.751	0.75
8	5 (4–5)	4.62 (1.1)	0.4	0.7	2.4	11.4	20.6	44.7	18.0	1.8	0.800	0.77
9	4 (3–5)	3.97 (1.6)	4.0	4.4	8.8	15.6	18.9	35.7	11.9	0.6	0.669	0.66
									Eigenvalue		6.10	

**Table 2:** Data completeness (*n* = 544), unidimensionality, scaling assumptions, targeting and reliability (*n* = 520)

Property	UWES-9	Comments
Data completeness		
Missing item responses (minimum-maximum %)^a^	0.4–1.8	^a^ Should be < 5%
Computable scale scores (%)	95.4	
Scaling assumptions		
Corrected polyserial item-total correlation	0.66–0.89	
Unidimensionality		
Factor loadings (min-max) in Factor 1	0.669–0.913	
Factor 1/Factor 2% common variance explained	72.4/9.5	
Factor 1/Factor 2% common variance explained in Parallel analysis ^b^	27.1/23.1^c^	^b^ Parallel analysis based on the 95th percentile of each factor’s percentage of common variance explained from a permutation of 500 random polychoric correlation matrices. ^c^ The variance explained by the random model (23.1%) exceeds that explained by the empirically observed data (9.5%), suggesting no support for the presence of a second factor in this case.
Targeting		
Possible score range (midpoint)^d^	0–6 (3)	^d^ Higher values indicate better self-reported work engagement.
Mean (SD) score^e^	4.42 (0.9)	^e^ Should be close to the scale midpoint.
Median (q1-q3) score^e^	4.67 (3.89-5.0)	^e^ Should be close to the scale midpoint.
Minimum-maximum score^f^	1.0–6.0	^f^ Should span most of the scales score range.
Floor effects (%) ^g^	0	^g^ Should be < 15%
Ceiling effects (%)^g^	2.1	^g^ Should be < 15%
Skewness^h^	-0.74	^h^ Should be between − 1 and + 1
Reliability		
Ordinal alpha^i^	0.93	^i^ Should be *≥* 0.70
Ordinal alpha if item deleted (minimum-maximum)	0.92–0.93	

## Discussion

In the present study, the psychometric properties of the UWES-9 among Scandinavian haemodialysis nurses were assessed using data collected prior to the COVID-19 pandemic. To the best of our knowledge, this is the first methodological study to account for the ordinal nature of the scale’s item-level data. In this context, it becomes challenging to directly compare our results with those of studies employing the same methodology. The classical-test-theory-based observations conducted indicate general support for a 1-factor structure of the UWES-9. These results align with previous findings among Swedish working women [10] and healthcare workers in Hong Kong [30], but contradict to the originator’s results [7, 31] and recent findings among Spanish healthcare workers [32]. These variations may stem from methodological differences, such as sample characteristics or analytical approaches, as well as contextual factors influencing responses. Understanding these discrepancies is crucial, as they may inform refinements in the measurement and application of the instrument. Future research should further investigate these differences to assess their generalisability and practical significance. As exemplified by the aforementioned Spanish study which was influenced by the ongoing COVID-19 pandemic, a Dutch study showed a marked reduction in work engagement ratings [15]. Moreover, the Spanish study employed tests that did not account for the ordinal nature of the data. Taken together, these aspects suggest that the Spanish study’s results are probably not relevant to normal (non-pandemic) working conditions. Because the UWES-9 ratings were considered to violate one important assumption –that they were measured on an equal interval scale –a traditional Pearson correlation matrix would underestimate the relationship between the items [16]. Therefore, analyses were conducted using polychoric correlations. To minimise potential bias arising from variations in working conditions, a homogeneous sample comprising a single nursing speciality was selected, as factors influencing work engagement — such as emotional stress, physical demands, and professional satisfaction [33] —, can vary considerably across nursing specialties.

Data completeness was high, with a very low proportion of missing responses across items. This is widely regarded as an indicator that the scale is both comprehensible and user-friendly for respondents. The findings lend support to the legitimacy of aggregating item scores into a total unidimensional score, suggesting that the scale captures a coherent underlying construct. Although the UWES-9 item scores were somewhat skewed towards higher values, targeting — that is, the alignment between item difficulty and respondents’ levels of work engagement — was broadly consistent with expectations based on distributions reported in previous multinational, multi-occupational samples [34]. This indicates that the scale functions similarly across diverse populations, supporting its generalisability. Furthermore, ceiling effects were minimal, and no floor effects were observed, which is favourable from an outcome assessment perspective, as it implies that the scale retains sensitivity across the range of engagement levels without clustering scores at the extremes. However, the observed positive skewness may challenge the interpretability of mean scores, as it suggests that the mean may not adequately represent the central tendency of the data. This issue underscores the need to consider the median as an alternative measure of central tendency, particularly when summarising or comparing groups in future studies.

The internal consistency (reliability indices) exceeded recommended thresholds slightly, a finding which may reflect a degree of item redundancy or the presence of highly similar items [22]. While redundancy can, in certain instances, signal opportunities for scale refinement, in this context the retention of all items is theoretically defensible. Each item represents a distinct yet interrelated dimension of the underlying construct, thereby enhancing the comprehensiveness and internal consistency of the scale. Eliminating items solely on the basis of redundancy could risk the exclusion of conceptually significant content. Nevertheless, the potential value of future research aimed at examining possible refinements—particularly through qualitative validation and advanced psychometric methodologies—is duly acknowledged.

Given that prior studies have reported Cronbach’s alpha coefficients, direct comparisons of internal consistency indices are precluded as the present study employed ordinal alpha. Although conceptually analogous, ordinal alpha has been demonstrated to yield more accurate reliability estimates for ordinal response scales [35]. Accordingly, the present findings lend support not only to the acceptability of the scale but also to its underlying psychometric properties. While these observations must be confirmed in other samples of nurses, we recommend the continued use of the UWES-9 as a unidimensional instrument for assessing work engagement.

### Limitations

While the present study provides valuable insights, it also has limitations. The cross-sectional sampling procedure used may limit the generalizability of the findings because the data reflect a snapshot and may not extend beyond the specific context and sample studied. Similarly, only including nurses specialized in haemodialysis care might have limited the study’s representativeness of nursing population estimates. Replication studies with large samples from various nursing specialties, allowing for comparisons of psychometric properties, are therefore desirable. Another limitation of the study is that we used two existing translations (Danish and Swedish) of the UWES-9 questionnaire without ensuring cross-language equivalence, which may have introduced some cultural or contextual biases into the data that could have influenced the factor structure. Future research should consider conducting validation studies across different languages and cultural groups to ensure that the scale functions equivalently across contexts and to mitigate potential biases arising from language-specific interpretations. There is also a slight difference in the working conditions between Danish and Swedish haemodialysis nurses, with nurses in Denmark having a slightly shorter workweek, which may be reflected in the reported levels of work engagement. There is a negligible possibility that this phenomenon may have introduced a form of response bias. Finally, relying solely on EFA has certain limitations. In particular, EFA does not allow for the confirmation of a hypothesised factor structure. We recognise that a confirmatory factor analysis would provide further validation and support for the factor structure identified. As such, we recommend that future research should incorporate confirmatory factor analysis to confirm the factor structure and test the robustness of the findings across different samples.

### Implications for nursing management

Through their leadership, nurse managers and leaders are key players in promoting work engagement in the organization. As greater engagement among nursing staff can lead to improved job satisfaction, improved patient safety, and reduced turnover, a more positive work environment can be advanced by using tailored interventions, such as targeted training programmes, team-building activities, and workload adjustments. The UWES-9 is a structured, widespread and accepted method of assessing engagement levels; however, the present study provides valuable and new insights into how managers and leaders should use the UWES-9 appropriately. In contrast to the originator’s intention, the UWES-9 is better employed as a unidimensional scale to evaluate nurses’ work engagement.

## Conclusion

The study findings, evaluated using methods that consider the ordinal nature of the scale ratings, indicate that the UWES-9 is a reliable and valid unidimensional measure with high data completeness, strong internal consistency, and a supported factor structure. These results support the use of the UWES-9 in research and practice for assessing work engagement. However, further studies are recommended to examine its applicability across diverse populations and settings.

## Data Availability

Data cannot be shared openly to preserve the individuals’ privacy under the European General Data Protection Regulation and due to ethical approval restrictions.

## Abbreviations

EFA: Exploratory Factor Analysis
KMO: Kaiser-Meyer-Olkin measure of sample adequacy
MAP: Minimal Average Partial test
UWES: Utrecht Work Engagement Scale
UWES-9: Utrecht Work Engagement 9-item Scale
